# Does Scale-Free Syntactic Network Emerge in Second Language Learning?

**DOI:** 10.3389/fpsyg.2019.00925

**Published:** 2019-04-26

**Authors:** Jingyang Jiang, Wuzhe Yu, Haitao Liu

**Affiliations:** ^1^Department of Linguistics, Zhejiang University, Hangzhou, China; ^2^Institute of Quantitative Linguistics, Beijing Language and Culture University, Beijing, China; ^3^Center for Linguistics and Applied Linguistics, Guangdong University of Foreign Studies, Guangzhou, China

**Keywords:** syntactic emergence, complex network, dependency syntax, scale-freeness, small-worldness, second language learning

## Abstract

Language is a complex system during whose operation many properties may emerge spontaneously. Using complex network approach, existing studies have found that, in first language (L1) acquisition, syntactic complex network featuring the scale-free and the small-world properties, will emerge at the age of 24 months. For foreign language (L2) learning, however, researchers have not reached a consensus on whether syntactic network with these two properties will emerge. Therefore, this study adopts complex network approach in L2 learning study, attempting to answer this question. In this study, nine networks are constructed on the basis of English compositions by Chinese students. Properties of these networks reveal that the syntactic network featuring these two properties, instead of emerging suddenly at a certain point, has existed at the very beginning of the L2 learning of Chinese students, and persists throughout the entire process of L2 learning, which is different from what has been found in L1 acquisition. The reason is probably that the already established L1 syntactic system provides foundation for L2 syntactic learning, and L2 learners tend to use the entrenched L1 syntactic network to generate L2 syntactic structures. L2 syntactic learning thus is not characterized by a sudden emergence of syntactic system, but a gradual approximation to the target language, with its own unique properties. For the first time, this study provides a tentative answer to L2 syntactic emergence from the perspective of complex network, and provides a macroscopic description of L2 syntactic developmental trajectory.

## Introduction

The non-linear interaction of subsystems of a complex system usually results in an unpredictable outcome (Santos and Zhao, 2017), which is termed ***emergence***. It is defined as “the first systematic use of a structure” (Pienemann, 1984, p. 191), and involves “some new kind of relation” (Ellis, 1998, p. 631), in which “the whole is more than the sum of its parts” (Holland, 1998, p. 2). The phenomenon of emergence has become one of the most attractive subjects in the field of complex system.

Language is also a complex system (Liu, 2018), and language acquisition may be investigated from the perspective of complex system (Solé et al., 2010; Gromov and Migrina, 2017), complex dynamic system (Baba and Nitta, 2014; Wachs-Lopes and Rodrigues, 2016), and complex adaptive system (Beckner et al., 2009; Ellis, 2016; Lu et al., 2016). MacWhinney (2015) listed 19 important emergentist mechanisms of language, including proliferation, competition, generalization, error correction, self-organization, topological organization, etc. He also introduced some specific quantitative methods, such as Parallel Distributed Processing, Self-Organizing Feature Maps, and Dynamic Systems Theory, which were utilized to account for the emergence of language in previous studies. However, MacWhinney and O’Grady (2015) have not mentioned the complex network approach, which is another effective way to analyze and explain the emergent properties of human languages.

Some important properties of complex networks, like the scale-free and the small-world properties, are found to emerge in first language (L1) acquisition. L1 acquisition goes through holophrastic stage, two-word stage with rapid vocabulary growth, and fluent grammatical conversation phase that features steadily increased sentence length and exponentially increased syntactic types (Radford, 1990; Corominas-Murtra et al., 2009). The development of L1 syntax is non-linear: at a certain age, the growth may be explosive, which is what is meant by emergence. Resorting to empirical data and complex network approach, Corominas-Murtra et al. (2009) found, for the first time, that at the age of 24 months, two important complex network properties, i.e., the scale-free property and the small-world property, emerge in L1 syntactic networks, signaling a sharp transition from a pre-syntactic organization to a scale-free and small-world syntactic network. Therefore, the emergence of syntax, which concerns the change of word-word relations, signals the rapid shift from chaotic word clusters to well-organized sentences. From the perspective of complex network, the emergence of syntax means the shift from a non-scale-free and non-small-world language structure to a scale-free and small-world language network.

As for L2 syntactic learning, various studies have attempted to find out whether there is such an abrupt shift in L2 learning, but failed to reach consensus on this issue. L2 learning is viewed as a dynamic and complex process teeming with emergences (Larsen-Freeman, 1997; Ellis, 1998), and several theories are suggested to explain this process. Chaos/Complexity Theory, for instance, may uncover the patterns underlying complex and dynamic language system (Holland, 1998; Larsen-Freeman and Cameron, 2008). Dynamic Systems Theory (DST) views learner language as a continually changing system, takes into consideration all variables concerning learning, and tries to model this system mathematically (e.g., de Bot et al., 2007). In L2 development, emergences, or, sudden shifts, which reflect system restructuring, have already been observed by empirical studies. For example, Sato (1988) investigated the emergence of complex syntactic structures in L2 development of two Vietnamese learners of English, especially the coordination, the subordination, and the complement structures. Besides, the emergence of English verb-argument constructions, such as verb locative, verb object locative, and ditransitive, were examined in L2 development (Ellis and Larsen-Freeman, 2009), with the finding that the acquisition of verb-argument constructions was influenced by input frequency, prototypicality and generality of the semantic types. With DST methods, Spoelman and Verspoor (2010) collected 54 writing samples (spanning 3 years) of a Dutch learner of Finnish, and detected in them a sharp change in noun phrases and the competition between noun phrase complexity and sentence complexity during these 3 years. Mellow (2006, 2008) diachronically investigated into the language production of a 12-year-old Spanish learner of English, and found that the complex structures such as relative clauses and argument dependencies of verbs emerged from item-based learning.

However, these researches all have their limitations. For one thing, these investigations failed to elucidate the emergence of syntax. As discussed above, the emergence of syntactic system is signaled by a rapid shift from arbitrary word clusters to well-organized grammatical sentences, which have not been examined in above studies. For another, these studies are microscopic, usually involving only a few participants, or a few specific structures such as verb-argument constructions, relative clauses and argument dependencies of verbs. As a result, they may fail to provide a panoramic picture of L2 syntactic development.

Based on large-scale language materials, this study aims to macroscopically explore the overall syntactic development in L2 learning, by employing network approaches to measure syntactic changes. Corominas-Murtra et al. (2009, 2010) analyzed two complex network properties of child language: the scale-free and the small-world properties, identified for the first time the precise time of the syntactic explosion in L1 acquisition, and reported that at the age of around 24 months occurred a sharp transition from the pre-syntactic organization to the scale-free, small-world, heterogeneous syntactic network. Complex network approach is thus capable of capturing the overall features of a network, throwing light on the phase transition, and providing potent quantitative measures for system-level analysis (Cong and Liu, 2014). Hence, to capture a holistic picture, this study employs complex network analyses into English syntactic development of Chinese learners, from primary school years, through junior high school years, to senior high school years. The following two research questions will be addressed:

(1) From a macroscopic complex network perspective, is there an English syntactic explosion in Chinese L2 learning? Or rather, do the properties of scale-freeness and small-worldness emerge in syntactic networks of Chinese learners of English? What is the overall syntactic development trajectory of the Chinese L2 learners across the 9 grades?
(2) What are the differences in the properties of syntactic networks of the nine grades? And what differences in lexical and syntactic capacity of each grade are indicated by these differences in network properties?

Question 1 is aimed to grasp the most prominent overall features of the 9 syntactic dependency networks. Question 2 is intended to figure out the specific differences among different stages of syntactic development of L2 learners. It is hoped that the answers to these two questions can shed light on the L2 syntactic development from beginners to high-level learners, which could be of much significance to language pedagogy.

## Materials and Methods

### Background Information of Participants and the Corpus Construction

The participants are 509 Chinese students, whose grades range from P_4_ (fourth graders of the primary schools) to S_3_ (third graders of the senior high schools). These students are from two primary schools, two junior high schools, and two senior high schools. Every pair of schools have similar quality in education, the same English syllabus, the same English textbook, and the same class hours each week. So the learning environments are very similar and we can thus minimize the interference of irrelevant factors. The English teachers were consulted about the English proficiency of these grades. They are very sure that on the whole, the students’ English proficiency of a higher grade is better than that of a lower grade, though the English test paper for the higher grade is more difficult than that for the lower grade and the average score of the former is even a bit higher than that of the latter. Grades are believed to be one of the most valid indexes for language proficiency (Wolfe-Quintero et al., 1998; Lu, 2011). Besides, to eliminate the “outliers” of each grade, a simplified version of questionnaire based on LHQ 2.0 (Li et al., 2014) was employed, including questions about age, gender, education, years of English learning, after-school English learning courses, overseas experience, and self-evaluation of English level. Average-level students whose language proficiency were representative of their grades were then selected as participants according to the questionnaire and their English quiz scores. Hence, in this study, student grade, i.e., the years of English learning, was an acceptable proficiency indicator of English. Therefore, writings by students from P_4_ to S_3_ are used to observe whether there is an emergence of scale-free and small-world syntactic network during the process of second language learning. The information of each grade, including the age and years of English learning, is presented in Table 1.

**Table 1:** A brief profile of participants.

Grade	Age	Years of English Learning
P_4_ (fourth grade of primary school)	9–10	0–1
P_5_ (fifth grade of primary school)	10–11	1–2
P_6_ (sixth grade of primary school)	11–12	2–3
J_1_ (first grade of junior high school)	12–13	3–4
J_2_ (second grade of junior high school)	13–14	4–5
J_3_ (third grade of junior high school)	14–15	5–6
S_1_ (first grade of senior high school)	15–16	6–7
S_2_ (second grade of senior high school)	16–17	7–8
S_3_ (third grade of senior high school)	17–18	8–9

Writing tasks for all participants were assigned by their own English teacher in classroom tests at roughly the end of the semester in December. Students were fully aware that their compositions would be used for research, but their personal information would not be revealed under any circumstances. Written informed consent was obtained from participants above the age of 16, and the parents of all participants under the age of 16. The study was approved by the Research Ethics Board of Zhejiang University.

Students were required to finish their compositions in a prescribed period of time without any form of aid. Word count was prescribed (after consultation with the teachers in relevant schools) clearly for each grade in writing instructions: 40 words for fourth graders, 50 for fifth graders, 60 for sixth graders, 100 for the junior high school students and 130 for the senior high school students. In order to arouse the writing interests of the primary school students and to encourage them to write as much as possible, their topics such as “My Family,” “Fruit,” “Planting Trees” and “My Weekend” are presented in the form of colorful pictures; whereas for the high school students, the topics are presented in English, like “My Weekend,” “An Embarrassing Thing.” The topics include description and narration, because these two genres are not that demanding, and students are supposed to master these two genres at a very early stage of learning. The different topics are evenly distributed among the primary school students, but the junior and senior high school students used the same topics, respectively. By prescribing similar topics and genres, we intend to minimize the effects of irrelevant factors.

All the compositions were sent to us by their teachers, and were inputted into the computer after screening off the invalid ones, which are either too short (not meeting the minimal word count requirement of each grade), incomplete, irrelevant, or inappropriate. Besides, to ensure similar network sizes, which may facilitate the following comparative study, and guarantee consistency of the samples, it was decided that for each grade, the size of sample should be about 5000 word tokens. Therefore, 509 compositions were randomly chosen as the source of data for the current research, with a total of 45503 word tokens. Table 2 shows the information of the language materials, including the topic, the number of sampled compositions, and the number of word tokens of each grade.

**Table 2:** Information of the self-built corpus.

Group	Topic	No. of compositions	Word count
P_4_	Cleaning/Planting Trees/My Weekend/Clothes/Family/Fruit/My Classroom/	104	4909
P_5_	Cleaning/Planting Trees/My Weekend/Clothes/Family/Fruit/My Classroom/	91	5048
P_6_	Cleaning/ Planting Trees/My Weekend/ Clothes/Family/Fruit/ My Classroom/	70	5083
J_1_	My Weekend	57	5072
J_2_	My Weekend	49	5021
J_3_	My Weekend	36	5087
S_1_	A(n) Embarrassing/Surprising/Unforgettable Thing	37	5183
S_2_	A(n) Embarrassing/ Surprising/ Unforgettable Thing	32	5065
S_3_	A(n) Embarrassing/ Surprising/ Unforgettable Thing	33	5035

Total		509	45503

### The Construction of Syntactic Dependency Networks

In order to conduct complex network analysis, we have to construct language networks in the first place. However complex a network is, its basic elements are simple (Liu, 2008): vertices (or nodes) and edges (Newman, 2003, 2010). The former represents the entities of a network while the latter, the relationships between these entities. In a language network, vertices are linguistic units, such as words, and edges are pairwise relations between these linguistic units (Liu, 2008).

Suitable network representation is the prerequisite for the valid network analysis (Butts, 2009). To investigate the incremental syntactic development of L2 learners, syntactic networks are constructed, where a vertex represents a word type and the edge, the syntactic relation between two words. Besides, language networks must be constructed on the basis of linguistic rationales in order to be of research significance for language studies (Liu, 2011). Constituency, which concerns part-whole structures, and dependency, which concerns the binary asymmetrical relations between words, are two principal methods for syntactic analysis. In this study, the syntactic network is constructed on the basis of dependency structure. First, dependency grammar is shown to be more suitable for the research of language acquisition for learner language involving syntactic mistakes (Jiang and Ouyang, 2017). Second, the elements of dependencies are perfectly suitable to construct a network (Liu, 2008). Third, dependency grammar has been effectively applied to the investigation into L1 syntactic networks (Corominas-Murtra et al., 2009). For a reliable comparison between L1 and L2, dependency grammar is employed in the current study to construct syntactic networks.

**FIGURE 1:**
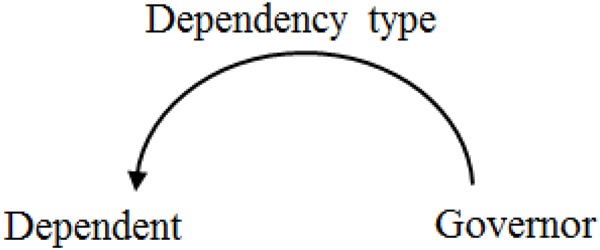
Three elements of a dependency.

**FIGURE 2:**
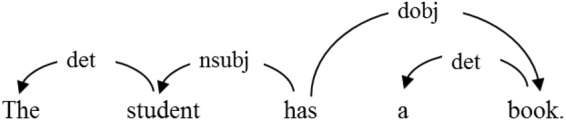
Dependency structure of the sentence *The student has a book*. *nsubj* is subject, *det i*s determiner, *dobj* is direct object.

According to dependency grammar, words in a sentence are connected by syntactic dependency relations (Mel’čuk, 1988; Hudson, 2010). In each dependency, there is a *governor —* the head word, and a *dependent* — the word governed by, or depends on the head word, as shown in Figure 1. The asymmetrical relation between the two words is shown by the arrow, which points from the governor to the dependent, with a label on the arrow to indicate the dependency type, or the syntactic relation. In Figure 2, for example, *The* depends on *student*; *has* governs *student* and *book*; *book g*overns *a*; and *has* is not governed by any word (i.e., *has* is the root of the sentence). From the perspective of network, the dependent and the governor in sentences are vertices, and the dependency relations between them are edges.

Dependency structures of sentences can be presented in forms of tables, as illustrated by Table 3, which gives the dependency structures of two English sentences (*This is a book* and *This book is very interesting*).

**Table 3:** Dependency structures of two sentences.

Order number of sentence	Dependent			Governor			Dependency type
	Order number	Word	POS	Order number	Word	POS	
1	1	This	DT	2	is	VBZ	nsubj
1	2	is	VBZ				
1	3	a	DT	4	book	NN	det
1	4	book	NN	2	is	VBZ	xcomp
2	1	This	DT	2	book	NN	det
2	2	book	NN	3	is	VBZ	nsubj
2	3	is	VBZ				
2	4	very	RB	5	interesting	JJ	advmod
2	5	interesting	JJ	3	is	VBZ	xcomp

*DT is a determiner, *NN* is a noun, *VBZ* is a verb in present tense, *RB* is an adverb, *JJ* is an adjective, *nsubj* is subject, *xcomp* is predicative, *advmod* is adverb modifier. In addition, all the punctuations have been deleted.*

**FIGURE 3:**
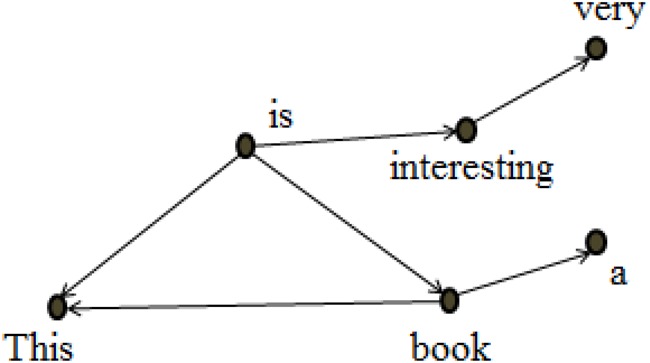
The syntactic dependency network of two sentences *This is a book* and *This book is very interesting.*

These dependency-annotated sentences can be converted into a syntactic network, as illustrated by Figure 3, which displays the syntactic network converted from Table 3. The vertices in the network are word types, and the directed edges among them indicate the asymmetric syntactic dependency relations. In Table 3, *a* and *This* depend on *book* which in turn depends on *is*. In Figure 3, hence, there are two edges pointing from the vertex *book* to *a* and *This*, and one edge pointing from the vertex *is* to the vertex *book*.

Syntactic treebanks are constructed through a syntactically tagging software Stanford Parser 3.6.0^1^ (Marneffe and Manning, 2008), and then are manually checked to ensure annotation accuracy. Details about this tagging software and the procedures are available in Ouyang and Jiang (2018). The syntactic errors in the students’ compositions are not corrected, and are left as they are in the treebanks as well as syntactic networks. The basic elements involved in network analysis are vertices and edges between them. One column of dependents plus another column of governors in the treebank will suffice for network construction. Therefore, syntactic errors, can only be reflected by the lack of edges between vertices in networks. For example, a learner made a syntactic error that the main verb *is* is missing in the sentence *This is a book*. Then, there would be no governor for the subject *This* and the object *book* in the treebank, and there would be no edge between vertices *is* and *This* and no edge between vertices *is* and *book* in the corresponding syntactic network. Take the sentence *I must go to home* as another case. The word *to* is redundant in this sentence, so there would be no governor for this word in the treebank and no edge linking to this vertex in the network. They will thus affect the network parameters such as degree and clustering coefficient. In this way, syntactic network can reflect language learners’ actual performance.

All the 509 compositions from nine grades were inputted into computer in plain text format, and each one was labeled with a unique code indicating the student’s school, grade, number and topic. Following the annotation principles of dependency grammar, nine syntactic dependency treebanks were automatically constructed through Stanford Parser 3.6.0, and then were manually checked. These dependency treebanks, each corresponding to a grade, were then converted into dependency networks by Createpajek^2^ (a network converting software, whose details are available in Appendix). So 9 syntactic networks are generated, each labeled according to the grade, such as network P_4_, network J_1_, network S_3_, and the like.

### Network Properties

Network analysis may produce a variety of quantitative measures (Newman, 2010; Liu, 2011) to characterize the macroscopic properties of language networks (Cong and Liu, 2014). In the present study, the differences in network properties among grades may reveal the cognitive mechanism underlying the process of language learning. In fact, network science has offered effective instrumentalities to capture complex properties of cognitive and behavioral processes. For example, cognitive scientists have adopted network theory to describe neural organization and cognitive processes (Baronchelli et al., 2013; Karuza et al., 2016), to explore human learning process (Karuza et al., 2017; Kahn et al., 2018), and to investigate language acquisition and learning (Sizemore et al., 2018). In this study, the macroscopic network properties like scale-freeness and small-worldness can be indicated by network parameters, such as degree, average degree, average path length, clustering coefficient. On the basis of these parameters, the emergence of network properties and its linguistic implications will be explored.

#### Degree

Degree (*k*) is a network parameter that measures the connectivity of a vertex in a network, referring to the number of edges which connect this vertex with others. In a directed network, the degree of a vertex may be classified into in-degree (centripetal) and out-degree (centrifugal) force, which represents a word’s ability to govern and to be governed by other words. In Figure 3, for instance, the degree of the vertex *book* is 3, with the in-degree being 1 and the out-degree being 2. In a syntactic network, the degree of a given vertex represents its syntactic relations with other words (Chen et al., 2011), and therefore measures its syntactic valency, i.e., its combinatorial ability to form syntactic dependencies (Cong and Liu, 2014). Hubs of networks are those vertices with high degrees.

*The average degree < k >* of a network is the mean of degrees of all its vertices, and thus may serve as an indicator of the connectivity of network. For example, there are 6 vertices in the syntactic network in Figure 3. The degrees of the 6 vertices *a, very, This, interesting, is*, and *book* are respectively 1, 1, 2, 2, 3, and 3, so the total degree is 12, and the average degree is 2 (12/6).

#### Clustering Coefficient

In a language network, the words linking to a given vertex may link to one another themselves. For instance, in Figure 3, the vertex *This* is linked to both *is* and *book*, which are also connected to each other themselves. *Clustering coefficient* is a network parameter reflecting the probability that two words linking to a vertex connect to each other (Newman, 2010). It measures the degree to which vertex gather together, that is, the transitivity of a network.

If vertex *i* is connected with *k_i_* vertices through *k_i_* edges, then the maximal number of edges among these nodes is *k_i_ (k_i_ -1)/2*. Suppose *E_i_* is the number of edges actually existing among the *k_i_* vertices, then the clustering coefficient *C_i_* of vertex *i* is the ratio of the actual number *E_i_* to the number of all possible edges, i.e.

$$\begin{array}{c} C_{i}=\frac{2E_{i}}{k_{i}(k_{i}-1)} \end{array}$$

The clustering coefficient of a network (*C*) is the mean of clustering coefficients of all its vertices:

$$\begin{array}{c} C=\frac{1}{N}\sum_{i=1}^{N}C_{i} \end{array}$$

In the syntactic network of Figure 3, the vertex *is* connects with 3 vertices, i.e., *This, book* and *interesting*, among which only *This* and *book* are connected. Therefore, the clustering coefficient of vertex *is* is 1/3, while for *very, a* and *interesting*, the clustering coefficient is 0.

#### Average Path Length

In a network, a vertex can combine with another vertex directly or connect to it via several other vertices. Thus arises the concept of *Path length*, which measures the number of edges between two vertices. The shortest path length is the degree of separation between two vertices. In Figure 3, for example, the shortest path length between *a* and *This* is 2, and between *a* and *interesting* is 3.

Consequently, the average path length *(L)* of a network is a parameter defined as the average of all the shortest path lengths in the network, calculated as:

$$\begin{array}{c} L=\frac{1}{\frac{1}{2}N(N-1)}\sum_{i>j}d_{ij} \end{array}$$

In this formula, *N* is the total number of vertices in the network; *d_ij_* stands for the shortest edges between vertex *i* and vertex *j*.

As for large-scale networks, the software Pajek^3^ (Batagelj and Mrvar, 2011; De Nooy et al., 2018) is used for calculating the above network parameters, and the procedures are introduced in Appendix.

#### Scale-Free and Small-World Properties

To examine the scale-free and small-world properties of a network, the comparison between the original graph and its counterpart random network has to be carried out. The corresponding random network, in this study, is generated automatically by Pajek with the same number of vertices and the same number of edges as the original one (Erdös–Rényi network, see the generation procedure in Appendix). In the random network, the connections among the vertices are random and vertices have the same probability to be connected.

The scale-free property of a complex network is concerned with the degree distribution for the network. As mentioned above, in a network, a vertex’s degree is the number of edges linked to it. Thus *P(k)* is the probability of a vertex to have a certain degree *k*. In a random network, the degrees of vertices follow binomial distribution or Poisson distribution (Newman, 2003, 2010). On the contrary, in a scale-free network, the degrees of vertices generally follow power-law distribution (Newman, 2003, 2010), which means that a small number of vertices have high degrees whereas many other vertices have low degrees. This power-law distribution can be formulated as:

$$\begin{array}{c} P(k)\sim k^{-y} \end{array}$$

The degree distribution follows the power law for some constant exponent γ, and then its corresponding cumulative degree distribution follows the Zipf’s law, with exponent γ’, and γ’ equals γ-1.

The small-world property is concerned with two network parameters: the average path length (*L*) and the clustering coefficient (*C*) (Newman, 2003, 2010; Ferrer-i-Cancho et al., 2004; Cong and Liu, 2014). Compared with its random network, a small-world network has almost small average path length and far greater clustering coefficient (Watts and Strogatz, 1998). This means that the vertices in a small-world network tend to cluster together locally, and the distance between any two vertices is not long. Therefore, such kind of network is believed to be the best for local and global communication (Watts and Strogatz, 1998).

Small-world and scale-free properties are important features of human language networks (Ferrer-i-Cancho and Solé, 2001; Ferrer-i-Cancho et al., 2004). Moreover, Corominas-Murtra et al. (2009, 2010) identified the emergence of these two properties in L1 acquisition of children, which may indicate a phase shift of children’s language from non-syntactic clusters to syntactic networks. Therefore, in order to examine whether a similar phase shift takes place in L2 learners’ immature language, this study investigates into the emergence of the scale-free and small-world properties in syntactic network of each grade.

## Results

Nine syntactic dependency treebanks are transformed into 9 syntactic dependency networks (P_4_, P_5_, P_6_, J_1_, J_2_, J_3_, S_1_, S_2_, S_3_), and the above parameters of each syntactic network are calculated. The results are presented in this section.

### General Information of the 9 Syntactic Networks

**FIGURE 4:**
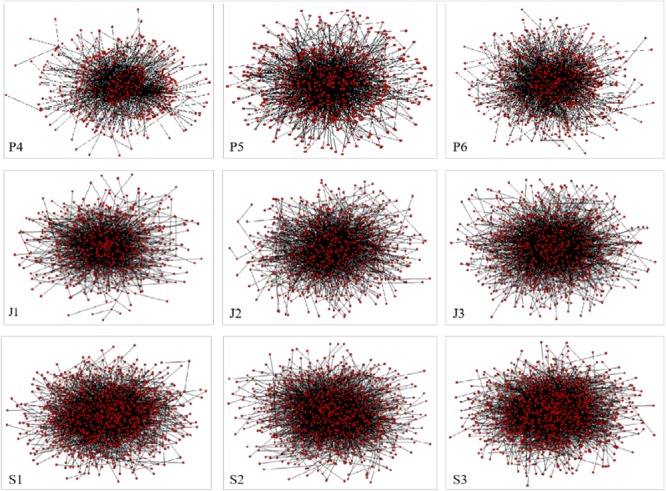
Nine global syntactic dependency networks (from P_4_ to S_3_).

Table 4 shows the general information of these networks from nine grades, including the number of vertices, the number of word tokens, and the Type/Token ratio (TTR). In these networks, vertices are word types, and the total number of words is the numbers of word tokens. To investigate lexical diversity/vocabulary richness of texts of similar size, the Type/Token ratio (TTR) is widely used (Richards, 1987). It is found that students from higher grades often present more lexical diversity or vocabulary richness. In addition, the global network of each grade (including all the vertices/word types) is presented in Figure 4 (see the drawing process in Appendix). Dots in graphs are vertices (word types), and lines among vertices are edges, i.e., syntactic dependency relations. The numbers of both vertices and edges increase steadily with the growth of grade.

**Table 4:** Lexical information of each network.

Network	P_4_	P_5_	P_6_	J_1_	J_2_	J_3_	S_1_	S_2_	S_3_
Vertices	551	644	671	661	711	800	1007	999	1121
Word tokens	4909	5047	5083	5072	5021	5087	5183	5065	5035
TTR	11.224	12.760	13.200	13.032	14.161	15.726	19.429	19.724	22.264

### The Scale-Free and the Small-World Properties of the 9 Syntactic Networks

**FIGURE 5:**
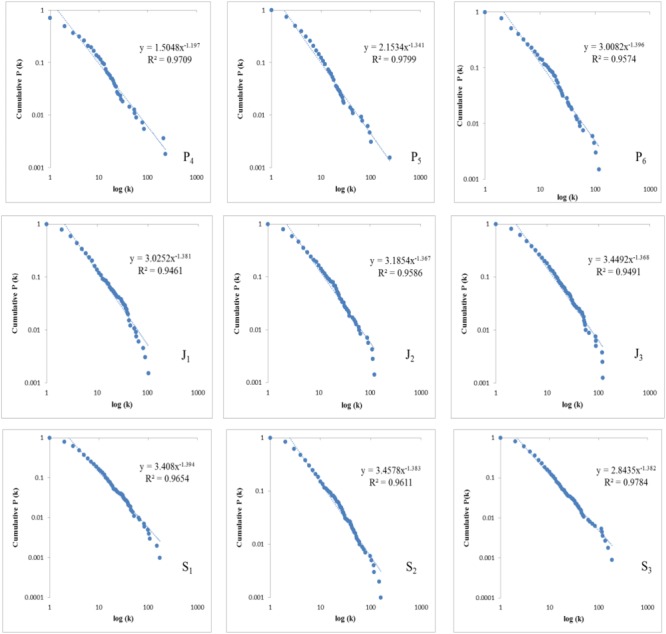
The cumulative degree distributions of 9 syntactic dependency networks.

To examine whether there is the emergence of scale-free property, the degree distributions of these 9 networks are extracted and compared with the degree distributions of their corresponding random networks (Newman, 2003), which may help identify the scale-free property of a network. For the purpose of reducing the noise in the long tail, the present study opts for the cumulative degree distributions (in log-log scales) of the nine networks, as shown in Figure 5.

**FIGURE 6:**
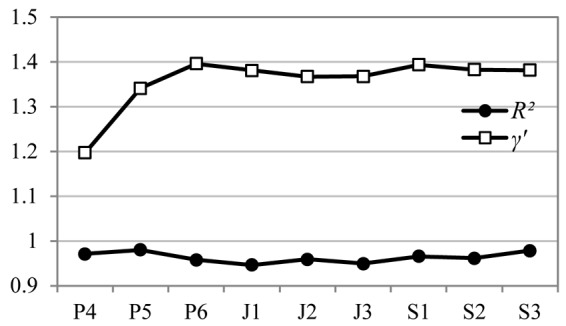
Power exponent γ’ and determination coefficient *R^2^* of nine syntactic networks.

It can be seen in Figure 5 that all the determination coefficients *R^2^* of nine syntactic networks are above 0.9, indicating that the degrees in 9 networks all fit well the power-law distribution, displaying Zipfian-like distributions. The fitting formulas are presented in each graph, with the power exponent γ’ and the determination coefficient *R^2^* presented in Figure 6, which registers a rapid increase of γ’ during the first three years of language learning, and then a tendency to stagnate at around 1.38 during the rest 6 years. The degree distributions of nine corresponding random networks are well fitted to binomial distribution, which is the essential feature of random Erdös–Rényi networks, and won’t be presented here due to the space limit. The Zipf’s law-like distributions of degrees in nine syntactic networks and the binomial distributions in 9 random networks suggest that the syntactic networks of all 9 grades exhibit a scale-free property.

Two network parameters, average path length (*L*) and clustering coefficient (*C*), are used to identify the small-world property of networks. Table 5 displays the average path lengths (*L*) and the clustering coefficients (*C*) extracted from the 9 syntactic network and the corresponding random networks.

**Table 5:** Major parameters of the nine networks and their corresponding random networks.

Networks	Word tokens	*N*	*< k >*	*C*	*L*
P_4_	4909	551	5.967	0.356	2.844
Random P_4_	4909	551	5.967	0.009	3.731
P_5_	5047	644	5.950	0.333	2.797
Random P_5_	5047	644	5.950	0.008	3.820
P_6_	5083	671	6.342	0.274	2.970
Random P_6_	5083	671	6.342	0.009	3.735
J_1_	5072	661	6.260	0.115	3.308
Random J_1_	5072	661	6.260	0.010	3.747
J_2_	5021	711	6.819	0.145	3.160
Random J_2_	5021	711	6.819	0.007	3.650
J_3_	5087	800	7.128	0.138	3.183
Random J_3_	5087	800	7.128	0.008	3.624
S_1_	5183	1007	6.878	0.114	3.236
RandomS_1_	5183	1007	6.878	0.008	3.804
S_2_	5065	999	7.033	0.122	3.227
Random S_2_	5065	999	7.033	0.005	3.771
S_3_	5035	1121	6.401	0.108	3.266
Random S_3_	5035	1121	6.401	0.005	3.991

*N, the number of vertices, <*k*>, average degree; *C*, clustering coefficient; *L*, average path length.*

Compared with the random network counterpart, a small-world network presents smaller average path length and far greater clustering coefficient (Watts and Strogatz, 1998). As Table 5 shows, all the nine syntactic networks have far greater clustering coefficients and smaller average path length than the corresponding random networks. These results suggest that all the 9 syntactic networks display small-world property.

### Lexical and Syntactic Development of the Primary School Students

**FIGURE 7:**
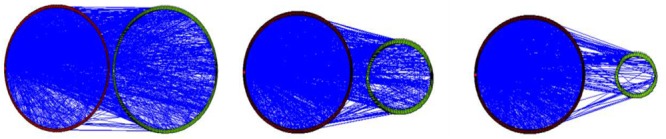
The Chinese–English syntactic dependency networks of P_4_ (on the left), P_5_ (in the middle), and P_6_ (on the right).

This section will be devoted to the English lexical and syntactic development of the primary school students, who have developed neither vocabulary nor syntax well enough to express ideas freely or construct sentences flexibly. Consequently, they had to rely on their mother tongue to finish the writing, and they were allowed (not encouraged) to occasionally use Chinese words. To faithfully reflect their actual use of English, Chinese words in the compositions of the primary schools students (P_4_, P_5_, and P_6_) are included in the analyses in the treebanks and the networks only in this section. The number and the percentage of Chinese word tokens in each treebank are presented in Table 6. The syntactic dependency networks of the three primary school grades are displayed in Figure 7. In each graph of Figure 7, for better visualization, English words are symbolized by vertices on the left circle, and Chinese words, by vertices on the right circle. The lines between the left and the right circles are syntactic dependency relations between English words and Chinese words.

**Table 6:** The use of Chinese words in the primary school students’ English compositions.

Grade	Chinese word tokens	Total word tokens	Proportion of Chinese
P_4_	1320	6229	21.19%
P_5_	391	5439	7.19%
P_6_	115	5198	2.21%

It can be seen in Table 6 and Figure 7 that in the primary school years, the use of Chinese words tends to decrease with the advancement of L2 learning. Chi-square test shows that the difference in percentage of Chinese words is statistically significant between P_4_ and P_5_ (21.19 vs. 7.19%, *p* = 0.008). Accordingly, the cross-language syntactic relations between English and Chinese words decreased with the grade growth, which are presented by the lines between the left and right circles in the graph. These results quantitatively reveal that the fifth graders (P_5_) have made considerable progress in their use of English, manifesting the importance of the first year in English learning.

### Comparisons of Network Parameters

A network has some important parameters, such as the number of vertices, average degree *< k >*, clustering coefficient (*C*), and the average path length (*L*), which can characterize the global network properties. As mentioned above, the primary school pupils often use Chinese words in their compositions. But when calculating these network parameters, Chinese words are excluded and only English words in the compositions are included for following network analysis, because the focus of our research is on the syntactic development of English.

**FIGURE 8:**
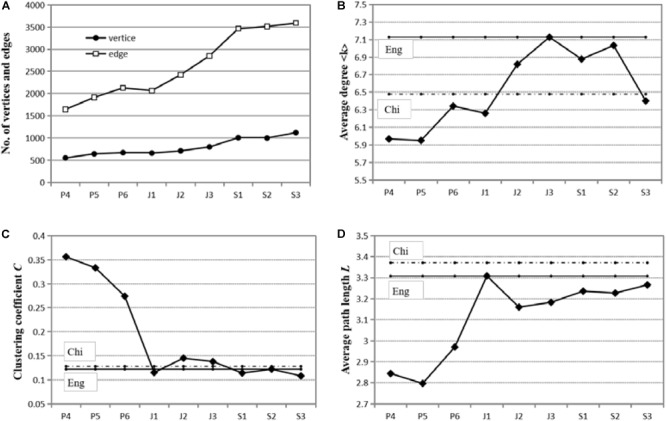
Changes in network parameters in L2 learning (from P_4_ to S_3_). Panel **(A)** shows the changes of number of vertices and edges from P_4_ to S_3_. Panel **(B)** shows the changes in average degree across nine grades. Panel **(C)** displays the evolution of clustering coefficient. The changes in average path length are shown in panel **(D)**.

The parameters of nine syntactic networks and nine random networks are presented in Table 5, and how these parameters change with the growth of grade is shown in Figure 8. The non-parametric Kruskal–Wallis test was employed to investigate whether there are significant differences between adjacent grades in these parameters. Figure 8A shows that the number of vertices monotonously increases with the growth of grade, and that the number of edges experiences a rapid growth when students move from J_1_ to S_1_. The changes in average degree are presented in Figure 8B, which shows that the average degree on the whole increases across the primary school and the junior high school years, except a slight decrease in J_1_, but fluctuates violently in the senior high school grades. Kruskal–Wallis test suggests that there are no significant differences in degree distribution between adjacent grades. Table 7 displays the 5 vertices (words) with the highest degrees in each network. It can be seen that the nine syntactic networks have similar high-degree words that function as hubs: *is/are/was, and, the, a, I, to, at*, and *in*. Among them, *is/are/was* has the highest degree (the most edges) in 6 networks, especially the networks of the primary school grades. For S_2_ and S_3_ learners, the preposition *to* has the highest degrees and becomes the most important hub of the network.

**Table 7:** The five vertices with highest degrees in the nine networks.

P_4_ Network		P_5_ Network		P_6_ Network	
**Degree**	**Word**	**Degree**	**Word**	**Degree**	**Word**
233	is	269	is	281	is
85	and	103	the	118	the
79	the	96	and	103	are
59	a	88	are	90	and
55	are	68	a	61	a

**J_1_ Network**		**J_2_ Network**		**J_3_ Network**	
**Degree**	**Word**	**Degree**	**Word**	**Degree**	**Word**
120	is	153	was	124	I
105	I	123	I	122	is
91	to	114	in	120	was
83	at	111	to	118	to
67	the	94	the	90	in

**S_1_ Network**		**S_2_ Network**		**S_3_ Network**	
**Degree**	**Word**	**Degree**	**Word**	**Degree**	**Word**

178	was	180	to	193	to
175	I	158	was	187	was
153	to	147	I	157	I
109	in	116	in	136	the
104	the	114	the	121	in

The changes of clustering coefficient (*C*) are displayed in Figure 8C. It can be seen that the clustering coefficient descends dramatically during the first 3 years, rises slightly in the junior high school years, and then fluctuates in the senior high school years. Kruskal–Wallis test suggests significant differences between the grades of P_4_ and P_5_ (*p* < 0.001), P_6_ and J_1_ (*p* < 0.001), J_1_ and J_2_ (*p* = 0.040), J_3_ and S_1_ (*p* = 0.003). Figure 8D shows the changes in average path length (*L*), which experiences a sharp increase in J_1_. Significant differences are found between the grades of P_6_ and J_1_ (*p* < 0.001), J_3_ and S_1_ (*p* = 0.010).

The language acquired by L2 learners, i.e., the “interlanguage,” is considered as closely related to both the native and the target languages (Selinker, 1969). Then it is valuable to compare network properties (parameters) of the interlanguage, the native language, and the target language, so as to pin down the possible unique features of interlanguage. The network parameters of native language (Chinese and English), which are from Liu and Li (2010), are presented in Figure 8B–D, where the full line indicates English network parameters and the dotted line indicates Chinese network parameters. The results of the comparison will be discussed in Section “Comparisons of Network Parameters of the Nine Networks.”

## Discussion

### Scale-Free and Small-World Properties of the Nine Syntactic Dependency Networks

Scale-free and small-world properties are two network indictors of the emergence of syntax. With the abrupt appearances of these two properties in L1 networks, Corominas-Murtra et al. (2009) identified the emergence of syntactic network in L1 acquisition. The present study also uses these two properties as network indicators, and the results show that the networks from nine grades (P_4_ to J_3_) all exhibit scale-free and small-world properties.

A scale-free network means that in this network a minority of vertices have extremely high degrees while a majority of vertices have relatively low degrees (Newman, 2003; Ferrer-i-Cancho et al., 2004). To recap, the degree of vertices follows a Zipfian-law distribution, as can be seen in Figure 6, which clearly points to the fact that the nine syntactic networks all exhibit the scale-free property. Obviously, this finding is different from the findings of L1 study by Corominas-Murtra et al. (2009), who reported that L1 acquisition experiences a phase shift from a non-scale-free structure to a scale-free network when children were about 2 years old. However, our findings show that there is no such phase shift in L2 learning.

But there are differences among nine networks in how well the Zipfian distribution fits the degrees of their vertices. These differences are reflected by the power exponent γ of degree distribution, which often ranges from 2 to 3 in a scale-free network (Barabasi and Albert, 1999). The γ’ of the corresponding cumulative degree distribution equals γ-1, so the value of γ’ is between 1 and 2. The more γ’ approximates to value “1,” the better Zipfian distribution fits the data. As can be seen in Figure 6, γ’ first rapidly increases during the first 3 years of language learning, then stagnates at around 1.38. In a syntactic network, the scale-free property reflects the different ability of vertices (words) to syntactically combine with other words (Cong and Liu, 2014). Therefore, the fact that the degree of a syntactic network fits Zipfian distribution better implies that the students are able to use these words more proficiently. One would assume that with the growth of age and grade, the syntactic network should produce exponent closer to value “1,” fitting Zipfian distribution better, which, however, is not confirmed in our study. This could be due to the large quantity of language mistakes made by the primary school students and the repeated use of similar sentence structures. Table 7 shows that in networks P_4_, P_5_, and P_6_, the top hub is invariably the vertex *is*, whose degree is constantly over 200, almost twice as many as the degrees of the top hub in networks S_1_, S_2_, and S_3_. In other words, networks P_4_, P_5_, and P_6_ display a more Zipfian-like distribution. However, the high degree of *is* in networks P_4_, P_5_, and P_6_ does not mean high language proficiency. Instead, it may result from the beginners’ limited lexicon and the repeated use of similar sentence structures such as *This is…* and *It is…*. A detailed explanation would be given in Section “Comparisons of Network Parameters of the Nine Networks.”

Compared with the random network counterpart, a small-world network has smaller average path length and far greater clustering coefficient (Watts and Strogatz, 1998). As Table 5 shows, all the nine syntactic networks display far greater clustering coefficients and smaller average path length than those of the corresponding random networks. These results suggest that all the syntactic networks display a small-world property.

Similarly, there is no abrupt emergence of small-world property during L2 learning. In other words, the syntactic networks of L2 learners exhibit small-world property even in the primary school years. In a nutshell, the dependency networks of L2 learners from all 9 grades display both scale-free and small-world properties. In contrast, such properties, which are absent at the initial stage, will suddenly emerge during L1 acquisition. This disparity can be attributed to the fact that during L2 learning, learners can rely on the syntactic similarity between the native language—Chinese and the target language—English in this case to construct sentences. It is known that both languages are SVO languages, with some slight differences, such as the placement of attributives, adverbials and the word order of interrogatives. For language beginners, most of their productions are simple sentences with basic subject-verb-object structure like *My family has six people. I have a brother. He is tall* and *The apple is haochi* (Chinese pinyin for *delicious*). These simple sentences have similar structures in both English and Chinese. Therefore, as long as the basic vocabulary is mastered, early English learners can readily construct English sentences by resorting to their syntactic knowledge of Chinese. Moreover, English dependency network has much in common with Chinese dependency network (Liu and Li, 2010). In other words, positive transferring of mother language’s syntax is possible, which may explain the absence of sudden emergence of syntactic complex networks in Chinese English learners. The syntactic knowledge of Chinese facilitates the mastering of English syntactic structures. Therefore, if native and target languages have similar syntactic structures, syntactic phase transition may not take place during L2 learning. This tentative conclusion, of course, needs more evidence from different languages. In addition, this lack of phase shift can be explained with the Unified Competition Model (UCM) proposed by MacWhinney (2012). There is a competition between native language and target language during L2 learning: the “entrenched” L1 knowledge constrains the learning of new knowledge of L2, and the degree of “entrenchment” depends on the extent of consolidation of the learner’s L1 (Li, 2015). As L1 knowledge becomes more solid, new and different L2 structures are more difficult to acquire. Chinese English learners begin to learn L2 at around the age of nine, and at that time their L1 syntax has already been well-established. Hence, learners will use the already well-established L1 syntactic rules to generate L2 sentences. L2 syntactic network, as a result, bears much resemblance to extant L1 network, which renders the phase shift very unlikely in L2 learning.

### Lexical and Syntactic Development of the Primary School Students

There are three reasons for the extra attention paid to the primary school students. First, as mentioned in Section “Background Information of Participants and the Corpus Construction,” Chinese students start to learn L2 at the fourth grade in the primary school, at the age of nine. L2 research has attached much importance to children’s second language learning (Lakshmanan, 1995). Therefore, this period is worthy of close examination: if there is the sudden emergence of syntax, which is signaled by the small-world and the scale-free properties, it may well occur in this period. Moreover, language immaturity and the resulting vast mistakes pose a great challenge for studies of primary school students’ English. Previous studies rarely investigate into the second language of children in this period, not to mention the syntactic development. Not least, different from students of high schools, the primary school students often use L1 in their compositions. Given these reasons, this section will closely examine the language progress in the primary school students.

For the primary school students, neither the L2 vocabulary nor the L2 syntax has developed well enough for them to express ideas or construct sentences freely in L2. Consequently, they have to rely on their L1 to finish the writing (Cohen and Brooks-Carson, 2001). This code-mixing phenomenon is the most prominent characteristic of L2 learning in this phase. It can be seen in Table 6 and Figure 7 that for the primary school students, the use of Chinese words tends to decrease with the advancement of L2 learning, as has been reported in previous studies (Wang and Wen, 2002; Woodall, 2002). Besides, the dramatic decrease of Chinese words at the grade of P_5_ is also noteworthy. As shown in Figure 7, the dependency relations between L1 words (the right circle) and L2 words (the left circle) decrease steadily with the grade growth. Most such dependency relations are replaced by English–English dependencies in networks P_5_ and P_6_. In brief, while L1 acquisition starts from scratch, L2 learning begins with the mixed use of two languages.

### Comparisons of Network Parameters of the Nine Networks

In order to explore the specific syntactic features exhibited at different stages of L2 learning, this section comparatively probes into the important network parameters of the 9 syntactic networks, including the number of vertices, the average degree *< k >*, clustering coefficient (*C*), and the average path length (*L*).

Table 4 shows the numbers of word types, tokens and TTRs of each network. It can be seen that the lexical diversity or vocabulary richness increases gradually with the grade growth. Figure 8A shows that the number of vertices increases steadily with the grade growth, and that the number of edges experiences, from J_1_ to S_1_, a rapid rise. Edges in a syntactic dependency network are syntactic relations between words. The rapid increase of edges thus indicates the rapid development of syntax. This means that the richness and complexity of syntactic relations, i.e., syntactic proficiency, have witnessed a substantial development in the junior high school year and the first senior high school year.

The changes in degree and average degree also deserve our attention. Vertices with relatively high degrees are hubs of a network. The 5 vertices with the highest degrees in the nine L2 networks are displayed in Table 7. L1 syntactic development witnesses a critical shift of hubs from semantic word *it* to function words *a* or *the* when the properties of scale-freeness and small-worldness emerge (Corominas-Murtra et al., 2009). However, in L2 syntactic development, the situation is different: all 9 syntactic networks have similar hubs (*is/are/was, and, the, a, I, to, at*, and *in*). Function words such as *and, the, a* have high degrees and play important roles even in the networks of L2 beginners. In addition, the copula *be* persistently has the highest degrees almost throughout the learning process. During the senior high school years, *to* has the highest degrees, becoming the most important vertex in networks. These findings are consistent with previous findings that function words such as articles and prepositions tend to be hubs of a network (Ferrer-i-Cancho and Solé, 2001; Cong and Liu, 2014; Jin and Liu, 2016). These hubs, i.e., the copula *be* and other function words, exists even in network P_4_, which may account for the absence of the phase shift, that is, the sudden emergence of scale-free property in L2 learning. Function words are key elements in sentence structures (Klammer et al., 2010). The high degrees of function words indicate their great combinatorial capacity in syntactic networks.

The average degree, as shown in Figure 8B, presents an increasing trend over the period from P_4_ to J_3_, except for a slight decrease in J_1_, and a descending trend in the senior high school years. The degree of a vertex (word) indicates its syntactic valency, i.e., its ability to form syntactic dependencies with words (Chen et al., 2011; Cong and Liu, 2014). Thus the said increasing average degree means enriched syntactic valency. That is, during the years of the primary school and the junior high school, students’ syntactic capacity develops gradually with the grade. However, during the senior high school years, the average degree firstly rises moderately, but then falls abruptly in S_3_, which is beyond our expectation. Probably, for Chinese S_3_ students, English learning has become a kind of repetitive practice and exercises for college entrance examination, resulting in the stagnation of syntactic development. As for the networks of mother tongues, the average degree is 7.127 for English and 6.478 for Chinese. Interestingly, the average degrees of networks J_2_, J_3_, S_1_, and S_2_ fluctuate within this range, somewhere between the average degrees of the native and the target languages. In other words, the interlanguage, namely the English acquired by Chinese students, is syntactically different from the target language. The disparity between interlanguage and target language is also revealed by the slight differences in the probability distribution of dependency distance (Ouyang and Jiang, 2018).

The changes in clustering coefficient are linguistically significant as well. As displayed in Figure 8C, the clustering coefficient descends dramatically during the first 3 years, rises slightly in the junior high school years, and then fluctuates in the senior high school years. The decrease of clustering coefficient in the first 3 years, especially in J_1_, can be attributed to the rapid increase of vertices, i.e., vocabulary, and the resulting “dilution” of edges, which may be construed as the lagging development of syntax. Note that the clustering coefficient (*C*) of a vertex is the ratio of the actual edges to all possible edges among its neighbor vertices. Hence, if a vertex links to only one vertex (i.e., poor combinatorial capacity), the *C* of this vertex is zero. The sudden drop in J_1_, therefore, implies that many vertices may have zero clustering coefficient, owing to the rapid increase of vocabulary and the simple syntactic structures used by beginners. In other words, J_1_ students may experience an expansion of vocabulary, but lack the ability to use these words creatively in new contexts. But with the advancement of learning, this ability gradually improves, leading to enriched syntactic valency and increased clustering coefficient. Verspoor et al. (2012) proposed that the learning focus for beginners is the accumulation of vocabulary, and given that the vocabulary threshold is reached, middle-level learners will shift much attention to sentence structures. Besides, networks of native English and native Chinese display quite similar clustering coefficient: 0.122 and 0.128 respectively. The clustering coefficient of the interlanguage, except for the primary school students, generally fluctuates around that of native languages.

The average path lengths (*L*) of these syntactic networks are shown in Figure 8D. *L* measures the network distance and the separation degree between a pair of vertices. As can be seen in Figure 8D, the average path length experiences a sharp increase in J_1_. Interestingly, the changing trajectory of average path length seems to inversely mirror that of clustering coefficient. The reason for the decrease of *C*, i.e., the rapid increase of vocabulary, may also account for the sudden rise of *L* in J_1_. When all vertices are linked to one center node, the *L* is the smallest, and the small value of *L* in a network has much to do with hubs (Watts and Strogatz, 1998; Nishikawa et al., 2002). Therefore, in networks P_4_, P_5_, P_6_, the relatively high degrees of the hubs, i.e., many dependency of words like *is*, lead to low *L*. The abrupt increase of *L* in J_1_ may also indicate that the syntactic ability lags behind lexicon, which has also been observed in L1 learners under the age of two, i.e., before the phase shift (Corominas-Murtra et al., 2009). Moreover, the boom of edges during the junior high school years (as seen in Figure 8A) may have much to do with the reduction of the average path length *L*. As to networks of L1, the average path length is 3.308 for English and 3.371 for Chinese. It can be seen that, for both junior and senior high school students, the average path length is slightly below those of both native English and native Chinese. This fact reflects the independence of the interlanguage to some extent. Similar to average degree, the average path length of interlanguage does not reach that of target language. This fact suggests that, even for advanced Chinese English learners, the L2 syntactic network is sparser than the network of native English, which means that it is not as well-organized as the syntactic network of native English.

From the above analysis, it can be concluded that in L2 learning there are no syntactic phase shift, namely, no sudden emergence of scale-freeness and small-worldness in syntactic networks of Chinese English learners. Different from L1 acquisition, which starts from scratch and features the sudden emergences of the above two complex network properties, L2 learning is usually based on an existing L1 syntactic network. Therefore, such an emergence is absent. Instead, there is a rather gradual approximation to the target language. Moreover, foreign language is acquired non-linearly and different linguistic subsystems (lexicon, syntax, etc.) are learned at different paces in different learning stages. Vocabulary goes through an expansion in the first year of the junior high schools, because in that year English becomes a major subject and textbook provides considerable vocabulary input. Syntax, on the other hand, does not substantially develop until late junior high school years and senior high school years, especially J_3_ and S_1_. In other words, syntax develops later than lexicon. In addition, the fluctuations of some parameters in senior high school years support the theory of fossilization (Selinker and Lamendella, 1978). In other words, language competence of L2 learners does not improve continuously with grade. Instead, after a certain period, the progress may slow down, stagnate, or even decline. Besides, through the comparison of these network parameters of the interlanguage, the native language and the target language, it can be seen that interlanguage has its independent status, not simply a variety of the target language that is distorted by the mother tongue. It actually displays properties which are unique, peculiar to itself.

## Conclusion

Based on dependency treebanks, network analyses of syntax provide new insights into the overall complexity of human language, which is unavailable with traditional linguistic methods (Cong and Liu, 2014). In the present study, quantitative analyses of dependency syntactic networks of L2 learners have answered the proposed research questions we intend to explore. First, in L2 learning there is no phase shift from a pre-syntactic structure to a syntactic network featuring scale-freeness and small-worldness. From the very beginning of L2 learning, i.e., the P_4_ grade, the syntactic networks of L2 learners have displayed the scale-free and small-world network properties, which is different from what has been observed in L1 acquisition. The reason is probably that the existent L1 syntactic system has already provided foundation for L2 syntax learning, and the entrenched L1 knowledge constrains the learning of new L2 syntactic knowledge. L2 learners tend to use the already well-established L1 syntactic network to generate L2 syntactic structures, which results in the absence of sudden emergence of the above two network properties. L2 syntactic learning is characterized by gradual approximation to the target language. Second, in L2 learning, the vocabulary gradually expands with the growth of grade, but the primary school students tend to rely heavily on their mother tongue in English writing, involving frequent code-mixing. The use of mother tongue declines significantly in P_5_ and vanishes in J_1_. Furthermore, network parameters, namely, the average degree, the number of edges, the clustering coefficient, and the average path length, indicate significant increase of lexical diversity in J_1_, and lagging maturation of syntax in J_3_ and S_1_. The syntactic network of Chinese English learners, even the advanced ones, is not as well-organized as that of native speakers. Third, the analyses of network parameters also reveal the fluctuation or fossilization of syntactic capacity of the senior high school students. In other words, the developments of L2 lexical and syntactic capacity are neither linear nor unidirectional.

Language as a complex adaptive system involves the interaction, cooperation and also competition among its subsystems (e.g., L1 and L2, lexicon and syntax). Complex network approach employed in this study provides operable instrumentality to investigate the complex system of language. The L2 syntactic system, as discussed above, is not isolated or independent. Instead, during the L2 learning, learners, especially the beginners, frequently resort to their entrenched L1 syntactic knowledge to construct sentences. For L2 learners, L1 and L2 are closely related. What’s more, in L2 learning, the significant increase of lexical diversity in J_1_ and the lagging maturation of syntax in J_3_ and S_1_ indicate the competition between lexicon and syntax of a single language. This study is the first to apply complex network method to macroscopically investigate the emergence of L2 syntax, enriching the study of language complexity.

However, our research does have some limitations. The most obvious one lies in the compositions we used, which are collected from different groups of students and different schools. This fact means our research is actually a pseudo longitudinal research. Language learners of the same grade also have great individual differences, and some of them may have after-school English lessons. Therefore, though the grade is a relatively effective way to indicate language proficiency, it would be better to group students according to learners’ actual language proficiency. That being said, we did try to have some control over the students and the compositions. For example, the students were all chosen from the schools with similar education quality and rankings. The students selected are those of average performance in each grade, neither the best nor the worst, which may guarantee that the students are comparable. The compositions from each grade are written and collected at the same time point, which makes sure that between every two adjacent grades, the difference in English learning time is exactly 1 year. Of course, in future studies, it would be desirable that participants should be divided strictly by their language proficiency. Also, though complex network approach offers us a macroscopic picture of language structure and some insight into the dynamic cognitive process in L2 learning, further explorations are needed to clarify the relationship between the macroscopic complexity and microscopic linguistic features.

## Author Contributions

JJ and HL conceived and designed the study. JJ and WY collected the data and performed the statistical analysis. All authors contributed to the result interpretation and manuscript writing.

## Conflict of Interest Statement

The authors declare that the research was conducted in the absence of any commercial or financial relationships that could be construed as a potential conflict of interest.

## Footnotes

1 Stanford parser can be downloaded from https://nlp.stanford.edu/software/corenlp-backup-download.html.

2 Createpajek can be downloaded from http://vlado.fmf.uni-lj.si/pub/networks/pajek/howto/excel2pajek.htm.

3 Pajek provides the users with efficient analysis of large networks and can be downloaded from http://vlado.fmf.uni-lj.si/pub/networks/pajek/.

## Appendix

With the syntactic dependency network in Figure 3 as an example, we list the overall procedure for the analysis of a syntactic dependency network as follows:

(1) Treebank building. Our treebank takes the form of an Excel table, with one column as the dependents and another column as their respective governors.
(1) Data transformation. The data of an Excel form (an.xls file) should be transformed into a.net file before importing into the software **Pajek** by using the software **Creatpajek**.
(3) Data importation. Click “File → Network → Read” on the main window of **Pajek**, then the.net file was imported into the software **Pajek**.
(4) Random network generation. The number of vertices and edges of the original network are obtained by clicking “Info → Network → General” on the main window of **Pajek**. Click “Net → Random Networks → Total No. of Arcs → input the number of vertices and edges of the original network,” then the corresponding directed random Erdös–Rényi network is generated, whose arcs need to be converted into edges. Click “Net → Transform → Arcs- > Edges → All,” then the corresponding random network is obtained.
(5) Network analysis.
  Click “Net → Transform → Remove → Multiple lines/Loops” on the main window of **Pajek**, then the multiple lines and the loops of are removed from the network;
  Click “Draw → Draw” on the main window of **Pajek**, then click “Layout → Energy → Kamada-Kawai → Free” on the draw window of Pajek, we could obtain a macro picture of the network;
  Click “Net → Partitions →Degree → All” on the main window of **Pajek**, then the *degree* of every vertex in the network are calculated;
  Click “Info → Network → General” on the main window of **Pajek**, then the value of the network’s *average degree* could be reached;
  Click “Net → Vector → Clustering Coefficient → CC1” on the main window of **Pajek** to calculate the *clustering coefficient* of the network;
  Click “Net → Paths Between 2 Vertices → Distribution of Distance → From All Vertices” on the main window of **Pajek**, the shortest path length of the network is obtained.
